# Intensive Intervention Improves Outcomes for Chronic Obstructive Pulmonary Disease Patients: A Medical Consortium-Based Management

**DOI:** 10.1155/2022/6748330

**Published:** 2022-06-27

**Authors:** Shunjin Zhao, Lini Zheng, Maoxian Zhu, Yuexiang Shui, Xuxin Bao, Jun Zhao

**Affiliations:** ^1^Department of Respiratory and Critical Care Medicine, The Second Affiliated Hospital of Zhejiang University, Lanxi Branch (Lanxi People's Hospital), Zhejiang, China; ^2^Department of Respiratory and Critical Care Medicine, Zhejiang Medical and Health Group Hangzhou Hospital, Zhejiang, China

## Abstract

Chronic obstructive pulmonary disease (COPD) is a major cause of morbidity and mortality. Strategies involving multidimensional approaches for the treatment of COPD are needed. This study aimed to evaluate the efficiency of medical consortium-based management for COPD. Patients were grouped in accordance with whether the hospitals they went to were under the medical consortium. We enrolled 141 COPD patients in the management group and 147 COPD patients in the control group. There was no predetermined sex and disease severity inclusion or exclusion criteria. Patients in the control group were managed by standard care, while patients in the management group were managed with intensive medical intervention jointly by specialists in the hospital and general practitioners and healthcare workers in community health centers. There was no difference in the basal demographics between the two groups. The basal condition of the management group was worse than that of the control group, demonstrated by a higher CAT score and a lower pulmonary function index. Half-year intensive intervention decreased CAT score from 17.28 to 15.62 and the Barthel ADL index from 73 to 60 in the management group, which was associated with better pulmonary rehabilitation, pursed-lip breathing, oxygen usage, and medicine regularity. The benefits became more obvious after one-year intensive intervention in the management group. There was a difference in mMRC grades and smoking cessation between the two groups. This study shows that a one-year intensive intervention improves the patients' health status and pulmonary function, suggesting that our medical consortium-based management is effective in the treatment of COPD.

## 1. Introduction

Chronic obstructive pulmonary disease (COPD) is one of the leading causes of morbidity and mortality worldwide and represents a substantial economic, social, and healthcare burden [1–3]. In China, because of the rapid change in economic development and increased aging population, COPD was the fifth leading cause of death [2]. Furthermore, a nationally representative study of the prevalence of COPD in China showed that 13.6% of adults aged 40 years or older had COPD [1], indicating that this disease has become a major public health problem. Strategies aimed that the prevention and treatment of COPD are needed urgently.

COPD is characterized by poorly reversible airflow obstruction and an abnormal inflammatory response in the lungs. The main site of airflow obstruction occurs in small conducting airways that are <2 mm in diameter. Loss of lung elastic recoil (due to destruction of alveolar walls) and destruction of alveolar support (from alveolar attachment) is the main reason for the failure to produce the outcome by prescribing medication alone [4, 5]. Thus, multidimensional approaches are recommended. In China, regional medical consortiums were developed by different levels (provincial, municipal, county, and village) of medical service providers (hospitals and primary-level clinics) through vertical referral, or by the same level of medical service providers through horizontal referral of specialties [6, 7]. It takes the responsibility of providing integrated and continuous healthcare services, including disease prevention, diagnosis, treatment, nutrition, rehabilitation, nursing, and health management, to local residents. Currently, a regional medical consortium has been successfully implemented for the prevention of hypertension and diabetes mellitus [8, 9].

This study is a regional medical consortium-based trial involving 2 groups of subjects with COPD in Lanxi city, Zhejiang, China. One group of subjects was managed with intensive intervention jointly by specialists in the hospital and general practitioners and healthcare workers in community health centers under regional medical consortiums designed as management groups. The other group of subjects were managed by standard care in other regional medical facilities. The aim of this study is to evaluate whether medical consortium-based management in China is effective for the prevention of COPD, which can inform medical reform in other countries.

## 2. Methods

### 2.1. Study Participants

Subjects were recruited by their family physicians from 3 community healthcare centers in Lanxi city, Zhejiang, China, from January to December 2019. There was no predetermined sex and disease severity inclusion or exclusion criteria.

Inclusion criteria for COPD were the measurement of one second forced expiratory volume/forced vital capacity (FEV1/FVC) <70%). These criteria are in accordance with the criteria published by the Global Initiative for COPD.

Exclusion criteria were as follows: (1) inability to adhere to clinical intervention, (2) mobility impairment, and (3) mental diseases. (4) Other systemic illness affects the performance of this intervention program.

### 2.2. Study Design

**Figure 1:**
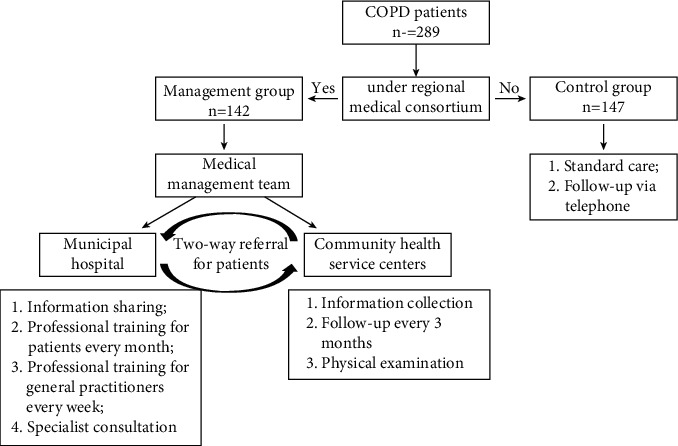
Study outline.

This is a community-based and parallel group trial involving 2 groups of subjects with COPD (Figure 1). Patients were assigned to the management or control groups according to whether the hospital they went to were under a regional medical consortium. One municipal hospital and three community health service centers were included in the regional medical consortium in Lanxi city.

In the control group, healthcare providers and general practitioners provided subjects with usual care according to the subjects' needs [10]. The content and frequency of usual care services were not standardized.

In the management group, the general practitioners and physical specialist were jointly in charge of the diagnosis and treatment of acute exacerbations, the formulation of maintenance treatment plan, monthly assessment of disease condition, and program adjustment. The specialists are responsible for specialist consultation, health education for patients every month, and professional training for general practitioners every week. The general practitioners were responsible for onsite follow-up, disease monitoring and evaluation, specialized health education, and medication guidance. Patients received telephone-based counseling and an assessment of their pharmacological treatment monthly. A specialist was consulted to assess and adjust the medical treatment for a deteriorating patient.

All patients were asked to attend a face-to-face follow-up visit to record their status at the healthcare stations every 3 months.

### 2.3. Data Collection

Age was calculated using date of birth and survey date. Gender (male or female), smoking status, frequency of pulmonary rehabilitation, purse-lip breathing, average oxygen use, and medication regularity were self-reported. Body mass index (BMI) was calculated by dividing body weight (kg) by height (m^2^).

### 2.4. Primary Outcome

The primary outcome used to evaluate subjects' health status was the COPD assessment test (CAT) score [11, 12]. It is a questionnaire designed to measure the impact of COPD on a person's life, and how this changes over time. GOLD-ABCD categories were calculated using the mMRC scale cutoff point ≥2.

### 2.5. Second Outcome

Secondary outcomes are changes in pulmonary function tests, as assessed by forced vital capacity (FVC), % predicted one-second expiratory volume (FEV1), % predicted FEV1/FVC, and mean expiratory flow at 75% (MEF 75), 50% (MEF 50), and 25% (MEF 25).

### 2.6. Barthel Index in ADL

The Barthel index (BI) is a commonly used measure of functional disability by quantifying 10 activities of daily living (ADL) for patients with COPD [13]. The total score is calculated by summing the response value to each of these items with a maximal score of 100. Scores ranging from 0 to 20 indicate full independence in physical functioning.

### 2.7. Self-Management

Self-management is defined as the active participation of patients in their treatment, which comprises distinct sets of activities [14]. In this study, taking medication, pursed-lips breathing, oxygen use, and smoking cessation were included as self-management activities.

### 2.8. Data Analysis

R version 4.0.2 and Python version 3.8 were used to conduct statistical analysis. The analytical methods were descriptive analysis (mean and standard deviation, or number and percent) and single-factor analysis (*t*-test, *χ*^2^ test, and analysis of variance).

## 3. Results

### 3.1. General Characteristics of COPD Patients

Between January, 2019 and December, 2019, a total of 288 patients (84% of whom were male) were assigned to either the management group (141 patients) or the control group (147 patients) (Table 1). The patients' ages ranged from 36 to 91 years, and the oldest patients had the longest course of disease, reaching 61 years. The first onset age of COPD ranged from 24 to 86 years. The baseline CAT level ranged from 2 to 32%. 97% of COPD were at mMRC grade ≥2. One second expiratory volume (FEV1) was 41% predicted based on the height and body weight. The groups were unbalanced regarding the highest education level (control vs. management: 3% vs. 8%), the percentage of the Barthel ADL index (0–20 points) (control vs. management: 39% vs. 52%), forced vital capacity (FVC) % predicted (control vs. management: 62% vs. 54%), and mean expiratory flow (MEF) at 25% (control vs. management: 1.46% vs. 1.17%), 50% (control vs. management: 0.78% vs. 0.63%), and 75% (control vs. management: 0.37% vs. 0.3%). The patient's assignment into GOLD-ABCD categories used mMRC scale cutoff point ≥2. The distribution (%) of patients in the ABCD categories were 0.68%, 36.05%, 2.72%, and 60.54%, respectively, in control groups; 2.84%, 26.95%, 0%, and 70.21%, respectively, in management groups. All 289 patients completed the trial.

### 3.2. Efficacy Outcomes

**Figure 2:**
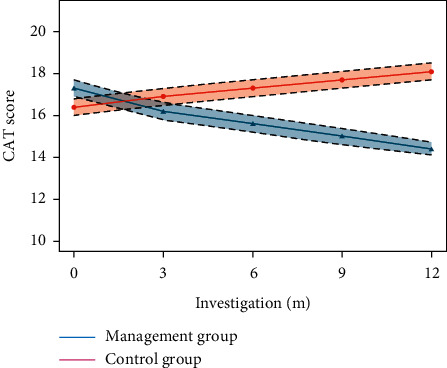
CAT in management group and control group in 12 months.

The mean CAT score decreased from 17.28 ± 4.47 to 14.45 ± 4.04 during the 12 months in the management group and increased from 16.41 ± 4.81 at baseline to 18.06 ± 4.62 in the control group (Table 2). The intervention effect was evident in 6 months and was consistent over 6 months (Figure 2). Both the percentage of patients with the mMRC grade ≥2 and the percentage of patients with the Barthel ADL index (0–20) decreased in the management group. Secondary outcomes are the pulmonary function indexes, including FEV1% predicted, FVC% predicted, and MEF at 75%, 50%, and 25%, respectively, which were increased in the management group and decreased in the control group in 12 months. Consistently, the distribution (%) of patients in high symptom groups (B and D) did not change in the control group while significantly decreased in the management group after 12-month intervention. Meanwhile, the fulfillment of core management skills by patients increased in the management group. The effects were evident in 6 months for pursed-lips breathing, oxygen use, and regular medication, and in 12 months for pulmonary rehabilitation. More patients quit smoking in the management group in 12 months.

### 3.3. Correlation between CAT and Self-Management Skills

**Figure 3:**
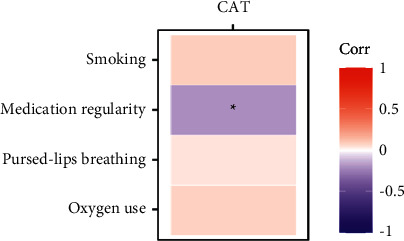
Correlation of CAT with self-management skills.

A strong positive correlation was detected between CAT with pulmonary rehabilitation and medication regularity (Figure 3). There is no correlation between CAT and pursed-lips breathing, oxygen, and smoking.

## 4. Discussion

In this study, we developed a medical consortium-based health management program and tested its potential as a reliable and effective community-based intervention strategy for the management of COPD in China. Although basal CAT and pulmonary functions in the management group are lower than those in the control group, one-year intensive management improved these indicators in patients.

### 4.1. Medical Care under a Regional Medical Consortium

The goal of health management for chronic disease management is to direct the limited resources available to patients with the greatest need for improvement [15–17]. Addressing chronic disease is a major challenge for healthcare systems around the world [18, 19]. Management of chronic diseases by grouping medical resources was first exemplified in the United Kingdom through the integrated care network, which aims to enable more residents to enjoy free medical services by setting up primary health centers and one-stop medical and social care services [20]. In the U.S., medical partnership systems started with the nonprofit medical insurance business of Kaiser Permanente and then evolved into integrated delivery networks. In this network, patients can enjoy an all-in-one service from the first visit through rehabilitation within the same insurance plan. China built regional medical consortiums in the second major healthcare reform since 2010. It aimed to provide healthcare services and healthcare management for residents by grouping medical institutes of different levels into teams with clearly defined responsibility [6, 7, 15, 21].

For patients with COPD, integrated multidisciplinary care is needed and has proven effective in improving the quality of life and health statuses of patients with COPD [22, 23]. In the north of the Netherlands, the asthma/COPD (AC) service supports general practitioners in managing patients with COPD [24]. Intervention for one-year has improved the quality of life in primary care COPD patients and their pulmonary functions with improved FEV1/FVC and mMRC [25]. Great efforts have been devoted to the management of COPD in China [26, 27]. This study found that community-based management for COPD showed an effective impact on the CAT score in patients. Furthermore, a moderate and persistent decline in pulmonary function and daily activity ability was observed in the control group, while an improvement in these indexes was observed in the management group in 12 months.

### 4.2. Self-Management Skills for COPD Patients

Behaviorally-based intervention in COPD was supposed to be effective [28, 29]. Self-management support is one of the essential components of the goal of the medical consortia. In this study, patients under the medical consortium got more intensive disease education and self-management support from general practitioners and physical specialists. In one-year health management, patients had increased medication regularity, average oxygen use, smoking cessation, pursed-lip breathing, and pulmonary rehabilitation. Correlation studies also showed that pulmonary rehabilitation and medication regularity were positively correlated with CAT. Thus, it is possible that these self-management skills might play a valuable role in supporting people with COPD to respond to changing symptoms and thereby make appropriate decisions regarding the management of their own chronic condition.

Smoking time and long-term oxygen therapy at home have been demonstrated to improve survival in patients with COPD [30, 31]. Our program showed significant effects on smoking cessation and oxygen use in one-year management. It is possible that the patients in the management group might have benefits in the future.

Rehabilitation serves as an important component of the management of COPD. Our data show that more patients in the management group performed pulmonary rehabilitation. COPD patients receiving oxygen therapy benefited from pulmonary rehabilitation than those COPD patients not receiving oxygen therapy [31]. It is suggested that pulmonary rehabilitation relieves dyspnea and fatigue, improves emotional function, and enhances the sense of control that individuals have over their condition [32, 33]. Thus, it is possible that the management intensive intervention might exert physical or psychological benefits on COPD in a longer follow-up trial [26].

### 4.3. Limitations

The main limitation of our study was the unbalanced regarding to the highest education level, the percentage of the Barthel ADL index, FVC % predicted, and MEF at 25%, 50%, and 75%, respectively, which made difficult to assess the impact of the interventions in the study group. However, patients in the management group had lower basal CAT, and pulmonary functions came to be improved more than those in the control group in our study. In addition, the number of patients was not large. A similar study with a larger sample should be conducted in the future.

## 5. Conclusion

Our community-based medical management effectively prevented the progression of COPD evidenced by decrease in the percentage of patients with the mMRC grade ≥2 and the percentage of patients with the Barthel ADL index and increase in pulmonary function indexes. Furthermore, there is a strong positive correlation between CAT with fulfillment of pulmonary rehabilitation and medication regularity.

## Figures and Tables

**Table 1:** Baseline characteristics of subjects.

Variable	Control group	Management group	*P*
Subjects, *n*	147	141	
Males, *n* (%)	119 (81)	123 (87)	0.147
Age (years)	73.73 ± 9.47	73.76 ± 7.89	0.846
BMI (kg/m^2^)	20.60 ± 3.44	21.06 ± 3.49	0.036
Highest educational levels (higher school and above), *n* (%)	5 (3)	12 (8)	0.019
Smoker, *n* (%)	72 (49)	67 (47)	0.805
First onset age (years)	59.04 ± 14.34	61.07 ± 12.68	0.281
Pulmonary rehabilitation	72 (49)	77 (55)	0.340
Pursed-lips breathing	23 (16)	26 (18)	0.530
Average oxygen use per day (hours)	1.17 ± 2.7	2.01 ± 3.49	0.031
Medication regularity	75 (51)	68 (48)	0.637
Barthel ADL index (≥60)	57 (39)	73 (52)	0.037
mMRC grades (≥2), *n* (%)	142 (97)	138 (97)	0.007
CAT	16.41 ± 4.81	17.28 ± 4.47	0.018
Pulmonary function			
FEV1/FVC (%)	55 ± 9.63	51.6 ± 10.21	0.004
FEV1/% predicted	45.17 ± 18.66	36.37 ± 15.31	2.190
FVC/% predicted	62.02 ± 19.44	53.77 ± 16.74	0.000
MEF25	1.46 ± 1.17	1.17 ± 0.95	0.006
MEF50	0.78 ± 0.6	0.63 ± 0.55	0.000
MEF75	0.37 ± 0.22	0.30 ± 0.19	0.000
GOLD-ABCD			
*A, n* (%)	1 (0.68)	4 (2.84)	0.208
*B, n* (%)	53 (36.05)	38 (26.95)	0.101
*C, n* (%)	4 (2.72)	0 (0.00)	0.123
*D, n* (%)	89 (60.54)	99 (70.21)	0.107

BMI: body mass index; Barthel ADL index: Barthel index for activity of the daily living scale; mMRC: modified British medical research council; CAT: the COPD assessment test; FVC: forced vital capacity; FEV1: one second expiratory volume; MEF: mean expiratory flow; other lung diseases: including bronchial asthma, bronchiectasis, tuberculosis, silicosis, and lung cancer.

**Table 2:** Differences in clinical characteristics between subjects of the control group and the management group after intervention.

Variable	Intervention for 6 months			Intervention for 12 months		
	Control group (*n* = 147)	Management group (*n* = 141)	*P*	Control group (*n* = 147)	Management group (*n* = 141)	*P*
Smoker, *n* (%)	68 (46)	53 (38)	0.137	63 (43)	34 (24)	0.000
Pulmonary rehabilitation	78 (53)	92 (65)	0.036	77 (82)	97 (69)	0.004
Pursed-lips breathing	35 (24)	73 (52)	0.000	42 (29)	81 (57)	0.000
Average oxygen use per day (hours)	1.22 ± 2.68	2.84 ± 4	0.000	1.31 ± 2.72	2.99 ± 4.21	0.000
Medication regularity	62 (42)	97 (69)	0.000	51 (35)	113 (80)	0.000
Barthel ADL index (≥60)	84 (57)	60 (43)	0.003	93 (64)	36 (26)	0.000
mMRC grades (≥2), *n* (%)	144 (98)	132 (93)	0.037	140 (95)	127 (90)	0.000
CAT	17.25 ± 4.52	15.62 ± 4.18	0.001	18.06 ± 4.62	14.45 ± 4.04	0.000
Pulmonary function						
FEV1/FVC (%)	54.12 ± 8.95	53.54 ± 8.79	0.561	53.56 ± 9.4	55.28 ± 8.5	0.185
FVC/% predicted	59.95 ± 17.58	58.58 ± 16.31	0.537	56.88 ± 16.83	61.96 ± 16.93	0.013
FEV1/% predicted	42.84 ± 16.98	40.94 ± 14.99	0.497	40.27 ± 16.21	44.76 ± 15.9	0.011
MEF25	1.39 ± 1.1	1.27 ± 0.97	0.603	1.34 ± 1.05	1.35 ± 0.99	0.329
MEF50	0.73 ± 0.58	0.7 ± 0.55	0.806	0.7 ± 0.56	0.72 ± 0.52	0.111
MEF75	0.36 ± 0.24	0.33 ± 0.2	0.442	0.34 ± 0.23	0.34 ± 0.19	0.023
GOLD-ABCD						
*A, n* (%)	2 (1.36)	5 (3.55)	0.274	3 (2.04)	11 (7.80)	0.028
*B, n* (%)	49 (33.33)	37(26.24)	0.2	40 (27.21)	38 (26.95)	0.999
*C, n* (%)	1 (0.68)	5(3.55)	0.114	4 (2.72)	4 (2.84)	0.999
*D, n* (%)	95 (64.63)	94 (66.67)	0.804	100 (68.03)	88 (62.41)	0.325

## Data Availability

The data that support the findings of this study are available from the corresponding author upon reasonable request.
